# APMCG-1: a mountain-cultivated ginseng-derived glycopeptide alleviates cerebral ischemic injury by resisting oxidative stress, ferroptosis and inflammation via the Nrf2 signaling pathway

**DOI:** 10.3389/fphar.2026.1768692

**Published:** 2026-04-14

**Authors:** Yuci Wu, Meiting Wang, Xingyue He, Chong Yang, Xindan Ai, Bingxiao Li, Han Yang, Kaijin Mu, Yulin Dai, Lei Liu

**Affiliations:** 1 Rehabilitation Medicine Academy, Changchun University of Chinese Medicine, Changchun, China; 2 Jilin Ginseng Academy, Changchun University of Chinese Medicine, Changchun, China; 3 Integrated Chinese and Western Medicine, Changchun University of Chinese Medicine, Changchun, China

**Keywords:** ferroptosis, ginseng, inflammation, ischemic stroke, Nrf2, oxidative stress

## Abstract

**Background:**

APMCG-1 is a glycopeptide derived from the mountain-cultivated ginseng C. A. Mey (APMCG-1), and recent studies have demonstrated its neuroprotective effects. Concurrently, ferroptosis plays an indispensable role in the pathogenesis of ischemic stroke. Investigate the neuroprotective effects of APMCG-1 in mitigating cortical brain injury and neurological dysfunction by suppressing oxidative stress, ferroptosis, and inflammation via the Nrf2 pathway.

**Methods:**

Rats underwent middle cerebral artery occlusion (MCAO) model. APMCG-1 (20 mg/kg and 40 mg/kg, gavage, 14 days). CoCl_2_ and glucose-free Dulbecco’s Modified Eagle Medium induce hypoxia-glucose deprivation/reperfusion (OGD/R) in a PC12-BV2 co-culture model. APMCG-1 (12.5, 25, and 50 μg/mL, 24 h). The study investigated how APMCG-1 modulates oxidative stress, ferroptosis, and inflammation in rat cortical and neuronal cells via the Nrf2 pathway.

**Results:**

APMCG-1 inhibited oxidative stress, ferroptosis, and inflammation, protecting against cortical injury and neurological dysfunction in MCAO rats, as well as safeguarding cells under OGD/R conditions. These effects were abolished upon Nrf2 inhibition by ML385, indicating that APMCG-1’s neuroprotective actions might depend on Nrf2 signaling.

**Conclusion:**

APMCG-1 could mitigate cerebral ischemia-reperfusion injury by inhibiting ferroptosis through the Nrf2 pathway, exerting antioxidant effects, and suppressing inflammation, thereby establishing a foundation for potential therapeutic strategies.

## 1 Introduction

Ginseng, a botanical drug commonly used in Northeast Asia since ancient times, has recently shown particular benefits in the treatment of Ischemic stroke (IS) (Dai et al., 2020). Many scientists have conducted studies demonstrating that ginseng has neuroprotective effects, including antioxidant and angiogenic effects, against IS (Zhao et al., 2022; Baik et al., 2021). Notably, ginseng is known primarily for its ability to mitigate oxidative stress, a key factor in the neuronal damage caused by IS. Nevertheless, existing ginseng studies predominantly focus on ginsenosides, with limited investigation of other bioactive components and their specific mechanisms against ferroptosis in IS. A novel glycopeptide was isolated from step ethanol precipitations of alkaline protease-assisted extract from MCG residue (APMCG-1). This represents an underutilized resource with significant therapeutic potential (Li Z. et al., 2025; Liu et al., 2026). It has been shown that APMCG-1 has a protective effect against ischemic brain injury (He et al., 2024). However, no further studies have investigated the therapeutic mechanism by which APMCG-1 protects neurons in the context of IS.

The pathophysiology of IS involves multiple interconnected mechanisms, including oxidative stress, neuroinflammation, and regulated cell death (Long et al., 2025; Wei et al., 2025; Pawluk et al., 2024). Ferroptosis, an iron-dependent form of regulated necrosis driven by lipid peroxidation, has emerged as a critical contributor to ischemic brain injury (Yang and Stockwell, 2016). Concurrently, the Nrf2 pathway serves as a master regulator of cellular defense against oxidative stress and inflammation, with growing evidence implicating its dysfunction in stroke pathogenesis (Peng et al., 2025). Current therapeutic strategies for IS primarily focus on thrombolysis and neuroprotection. However, recombinant tissue plasminogen activator (rt-PA) has narrow therapeutic windows and hemorrhagic risks (Chen et al., 2024), while existing neuroprotective agents have failed in clinical translation due to single-target limitations and poor efficacy.

Nrf2 is a key antioxidant transcription factor encoded by the NFE2L2 gene (Nuclear Factor Erythroid 2-Like 2 gene) (Chu et al., 2017). The role of Nrf2 in IS is believed to be pivotal, as they regulate cellular function and disease progression by restricting the production of reactive oxygen species (ROS) and thereby attenuating oxidative stress (DeNicola et al., 2011). The increased expression of Nrf2 proteins leads to the suppression of ferroptosis and inflammation, thereby preserving the stability of the blood‒brain interface (Liu et al., 2024). Furthermore, Nrf2 proteins are involved in attenuating IS by influencing the activation or inactivation of various signaling cascade responses (Zhang, 2025; Li and Xing, 2024). Recent studies have shown that Nrf2 knockout mice are more hypersensitive to Lipopolysaccharide (LPS) than wild-type mice and that Nrf2 knockout mice have significantly increased expression levels of NO (Nitric oxide), IL-6 and TNF-α (Wu et al., 2021). In addition to its role as an essential regulator of lipid peroxidation, Nrf2 also modulates the levels of other protective genes (Chen et al., 2023; Xu et al., 2023), including ferroptosis genes such as GPX4, iron transporter proteins (SLC40A1), and solute carrier family member 48 member A1 (SLC48A1). Ferroptosis is characterized by reduced levels of GPX4, a key enzyme controlling the antioxidant system (glutathione system) (Liao et al., 2023). It utilizes glutathione (GSH) as an electron donor controlled by the transporter protein SLC7A11 in the cell membrane to participate in oxidative reactions, thus effectively protecting liposomes and cell membranes from oxidative breakdown (Jia et al., 2025).

Given the unmet clinical need for effective neuroprotectants and the limited understanding of non-saponin ginseng components, this investigation of APMCG-1 provides both mechanistic innovation and translational potential for IS therapy. Hence, our study was to explore the impact and underlying mechanisms of APMCG-1 on Nrf2-mediated pathways, including antioxidant function, the inflammatory response, and ferroptosis, in both cellular and animal models. The hypothesis posited that ginseng could promote Nrf2-mediated antioxidant, anti-inflammatory effects and anti-ferroptosis to exert inhibitory effects in IS by virtue of its capacity to.

## 2 Materials and methods

### 2.1 Animals

Male Sprague-Dawley rats (9 weeks old, 240–260 g, SPF grade) were provided by Liaoning Changsheng Biotechnology Co., Ltd. Rats were housed under controlled environmental conditions (temperature 22 °C ± 2 °C, humidity 50%–60%, 12-h light/dark cycle) with free access to standard chow and water. All rats were acclimatized for 7 days before surgery.

### 2.2 Drug preparation

MCG was obtained from Fusong County, Jilin Province, China. The voucher specimen number of MCG is 202401 and stored in Changchun university of Chinese medicine. The plant name has been checked with http://www.worldfloraonline.org. APMCG-1 was isolated from MCG ethanol-extraction residues as previously described (Li Z. et al., 2025).

ML385 is a specific and direct inhibitor of Nrf2 transcriptional activity (Ye et al., 2025) (Yuanye, China). ML385 was administered by intraperitoneal injection 30 min prior to each APMCG-1 gavage.

### 2.3 Cerebral ischemia‒reperfusion modeling and grouping

Focal cerebral ischemia was induced by the intraluminal filament occlusion method (Jin et al., 2014). Briefly, rats were anesthetized with 2% pentobarbital sodium (40 mg/kg, intraperitoneal). A middle cerebral artery occlusion (MCAO) monofilament [(diameter 0.26–0.28 mm, RWD Life Science Co., Ltd.)] was inserted into the internal carotid artery via the external carotid artery until mild resistance. After 2 h of ischemia, the monofilament was withdrawn to allow reperfusion. The success of the MCAO model was confirmed by 2,3,5-triphenyltetrazolium chloride (TTC) staining. Sham control rats were subjected to similar MCAO operation procedures but without occlusion (Sicard and Fisher, 2009). Penicillin sodium (4 × 10^4^ U/rat) was administered by intraperitoneal injection for three consecutive days post-surgery to prevent infection.

On day 3 post-MCAO, successfully modeled rats were randomly divided into six experimental groups (n = 10 per group): Sham group (saline 5 mL, gavage), MCAO group (saline 5 mL, gavage), Aspirin group (MCAO, aspirin 20 mg/kg, gavage), APMCG-1 20 mg/kg group (MCAO, APMCG-1 20 mg/kg, gavage), APMCG-1 40 mg/kg group (MCAO, APMCG-1 40 mg/kg, gavage), and APMCG-1 + ML385 group (MCAO, intraperitoneal ML385 30 mg/kg, gavage APMCG-1 40 mg/kg). Treatment lasted for 14 days.

### 2.4 Neurological function assessment

Neurological function was assessed using the modified Neurological Severity Score (mNSS) and the Bederson scoring system (Bieber et al., 2019) at day 4 (as baseline) and day 18 (as endpoint). The mNSS is a composite of the motor, sensory, balance and reflex tests and is assessed on a scale of 0–18 (normal score 0, maximal deficit score 18) (Garcia et al., 1995).

### 2.5 TTC staining

Following neurological assessment, the infarct area was evaluated using TTC staining. The rat brains were removed and sliced into coronal sections then incubated at 37 °C in a 2% TTC solution for 30 min.

### 2.6 Hematoxylin-eosin staining (H&E)

Brain tissues were fixed in 4% paraformaldehyde, embedded in paraffin. The sections were stained with hematoxylin and eosin, incubated at 56 °C for 1 h and then washed with deionized water.

### 2.7 Nissl staining

Brain tissues were fixed in 4% paraformaldehyde, embedded in paraffin. The sections were immersed in Nissl staining solution, incubated at 56 °C for 1 h and then washed with deionized water.

### 2.8 Immunofluorescence

After dewaxing, antigen repair was performed with Tris-EDTA antigen repair solution. After blocking nonspecific binding, anti-GPX4 (rabbit, DF6701, Affinity, China) and anti-Nrf2 (Affinity, AF0639, 1:100), were used as the primary antibodies, and the sections were incubated at 4 °C. Then, 488-labeled goat anti-rabbit (1:200, B100805, Wuhan Bicentennial Bio) and 488-labeled goat anti-mouse (1:200, B100803, Wuhan Bicentennial Bio) secondary antibodies were added, and the samples were incubated for 1 h at room temperature. The images were observed using a Thermo Fisher EVOS fluorescence microscope and analyzed in ImageJ software.

### 2.9 Immunohistochemistry

After dewaxing, the paraffin sections were blocked with 5% goat serum at room temperature for 20 min. The samples were then incubated overnight at 4 °C with primary antibodies against GPX4 (rabbit, DF6701, Affinity, China) and Nrf2 (1:1,000 dilution). On the following day, the sections were treated with HRP at room temperature for 30 min, followed by DAB staining for 5–10 min.

### 2.10 Real-time quantitative PCR

TRIzol reagent (Life Technologies, USA) was used to isolate the total RNA from the brains of rats. Next, the RNA was reverse-transcribed into cDNA by FastKing RT Kit (TIANGEN, Beijing, China). The RT-qPCR was executed with Super Real PreMix Plus (SYBR Green, TIANGEN) using the CFX Connect RT- qPCR Detection System (Bio-Rad, USA). Table 1 showed the primers of the genes.

**TABLE 1:** Primer sequences RT-qPCR.

Gene	Forward primer	Reverse primer
GPX4	ACG​CCA​AAG​TCC​TAG​GAA​GC	CTG​CGA​ATT​CGT​GCA​TGG​AG
SLC7A11	TGC​CCG​GAT​CCA​GAT​TTT​CC	CAG​ATT​GCA​AGG​GGG​ATG​GT
Nrf2	AAT​TCC​CAC​CGC​CAG​GAC​T	TCA​AAC​ACT​TCT​CGA​CTT​ACC​CC
IL-6	AGA​GAC​TTC​CAG​CCA​GTT​GA	AGC​CTC​CGA​CTT​GTG​AAG​TG
IL-1β	CAC​CTC​TCA​AGC​AGA​GCA​CA	CGGGTTCCATGAAGTCA
TNF-α	CTG​TGC​CTC​AGC​CTC​TTC​TC	ACC​TGA​TGA​GAG​GGA​GCC​CAT
β-ACTIN	TGC​TGA​CAG​GAT​GCA​GAA​GG	CGG​ACT​CAT​CGT​ACT​CCT​GC

### 2.11 Cell culture

PC12 neuronal cells and BV2 microglia were obtained from iCell Biotechnology Co., Ltd. in Shanghai, China. PC12 and BV2 cells were cultured at 37 °C with 95% relative humidity and 5% CO_2_. Both cell types were maintained in Dulbecco’s Modified Eagle Medium (DMEM) supplemented with 10% Fetal Bovine Serum (FBS) and 1% f Penicillin‒Streptomycin (P/S).

### 2.12 Transwell coculture system

For the coculture experiments, we employed a transwell culture system to simulate *in vivo* conditions (Zujovic and Taupin, 2003). PC12 cells were seeded in the lower chamber of 6-well plates at a density of 2 × 10^5^ cells/well and cultured for 24 h to allow attachment. BV2 microglia (1.5 × 10^5^ cells/well) were then seeded in the upper chamber of 0.4-μm pore Transwell inserts placed above the PC12 cells. The coculture was maintained in DMEM, supplemented with 10% FBS and 1% P/S.

### 2.13 Oxygen glucose deprivation (OGD/R) and APMCG-1 treatment

PC12 cells were cocultured with BV2 cells for 24 h. Chemical hypoxia was induced by 4-h exposure to 160 μM CoCl_2_ in glucose-free DMEM, followed by 24-h reperfusion in complete medium (Nam et al., 2016). After 24 h of reperfusion, cells were randomized to six groups (n = 6 wells/group): Control; OGD/R; OGD/R + APMCG-1 (12.5, 25, or 50 μg/mL); and OGD/R + APMCG-1 (50 μg/mL) + ML385 (30 μM, added 30 min before APMCG-1).

### 2.14 Viability assay

Following a 24-h treatment period, the supernatant was removed, and 100 μL of diluted Cell Counting Kit-8 (CCK-8) solution (Shanghai Yuanye Biotech) was added to each well. The plates were then incubated at 37 °C for an additional 2 h. The absorbance was measured at 450 nm using a Tecan Infinite M200 Pro multimode microplate reader (Männedorf, Switzerland) to quantify cell viability.

### 2.15 Apoptosis staining

Cells were stained with Hoechst 33342 (10 μg/mL) (Solarbio, Shanghai, China) or acridine orange/ethidium bromide (AO/EB) staining (Shanghai Yuanye Biotech) for 20 min in the dark and imaged using fluorescence microscopy (Thermo Fisher EVOS) for blue, green, and red fluorescence. The quantification of cell apoptosis was performed by flow cytometry using an Annexin V-FITC/PI apoptosis detection kit (Bio-Opto Technology Co., Ltd., Shanghai, China).

### 2.16 ROS staining

ROS of PC12 cells was performed in 6-well plates with 1 mL of 5 μmol/mL DCFH-DA (Biosharp, Labgic) solution added to each well. After incubation for 20 min in a 37 °C cell incubator, the cells were rinsed three times with phosphate buffer solution (PBS) and observed under a fluorescence microscope (Thermo Fisher EVOS).

### 2.17 NO staining

NO staining of BV2 cells was performed via the addition of 1 mL of 5 μmol/mL DAF-FM DA (Biosharp, Labgic) solution to each well of 6-well plates. After incubation for 20 min in a 37 °C cell incubator, the cells were rinsed three times with PBS and visualized under a fluorescence microscope (Thermo Fisher EVOS).

### 2.18 Fe^2+^ level assay

Fe^2+^ levels in PC12 cells and cortical tissue homogenates were quantified by measuring the absorbance at 520 nm using a kit (Nanjing Jiancheng Bioengineering Research Institute Co., Ltd.) according to the provided instructions.

### 2.19 Superoxide dismutase (SOD), malondialdehyde (MDA) and GSH levels

After the PC12 cells and cerebral cortex tissues were homogenized, the levels of SOD, MDA, and GSH were measured following the instructions of the respective kits (Shanghai Yuanye).

### 2.20 Determination of IL-6, IL-1β and TNF-α levels

The levels of IL-6, IL-1β, and TNF-α in the tissue homogenate or supernatant after BV2 cells lysis were measured using ELISA kits according to the manufacturer’s recommendations (Shanghai Jianglai Biotechnology Co., Ltd.).

### 2.21 Western blotting

Protein was extracted and quantified with a BCA protein assay kit (Biosharp, Labgic). Proteins were separated by SDS‒PAGE electrophoresis and transferred to nitrocellulose membranes. The membrane was blocked with 5% nonfat milk at room temperature for 1 h and incubated with primary antibody overnight at 4 °C. The membrane was incubated with an HRP-labeled secondary antibody at room temperature for 1 h. The bands were developed and analyzed. The following antibodies were used: GPX4 (rabbit, DF6701, 3423676, Affinity, China), GSS (rabbit, DF6214, Affinity, China), GCLC (rabbit, DF8550, 3429172, Affinity, China), HO-1 (rabbit, AF5393, 8513764, Affinity, China), NQO-1 (rabbit, AF5266, 1213810, Affinity, China), SLC7A11 (rabbit, DF12509, 1754218, Affinity, China), Nrf2 (rabbit, AF0639, 8055401, Affinity, China) and β-actin (rabbit, AF7018,7364449, Affinity, China). Anti-rabbit IgG (H + L)-HRP (rabbit, S0001, 6681429 Affinity, China) was then used to detect protein signals.

### 2.22 Dataset acquisition and analysis

We used gene expression profiling data from the Gene Expression Omnibus (GEO) database. The GSE22255 dataset comprising peripheral blood samples from 20 patients with CIS and 20 normal controls was then selected based on screening exclusion. R language was used to process and analyze the data to obtain the cluster analysis heatmap. A protein‒protein interaction (PPI) network was constructed using the STRING database. The PPI network was then imported into Cytoscape 3.8.2 for topological analysis to identify core targets and construct the core protein‒target network. The core targets were subjected to enrichment analysis using Gene Ontology (GO) and Kyoto Encyclopedia of Genes and Genomes (KEGG) enrichment. R software was employed to convert the shared target gene names between diseases into specific gene IDs. A significance threshold of *p* < 0.05 was set for differential analysis. The GO analysis included three categories of gene function annotations: biological processes, cellular components, and molecular functions. The top 20 enriched functions in each category were selected. Similarly, the top 20 enriched KEGG pathways were identified using the same significance threshold (*p* < 0.05). Corresponding bar charts were generated for both the GO and KEGG enrichment analysis results.

### 2.23 Statistical analysis

Statistical analysis was done through GraphPad Prism 9.0 statistical software (GraphPad Software Inc., San Diego, CA, USA). Data were analyzed using a one-way ANOVA followed by Tukey’s multiple comparisons test and depicted as the mean ± SEM. *P <* 0.05 was considered significant.

## 3 Results

### 3.1 Pharmacological and physicochemical characterization of APMCG-1

**FIGURE 1:**
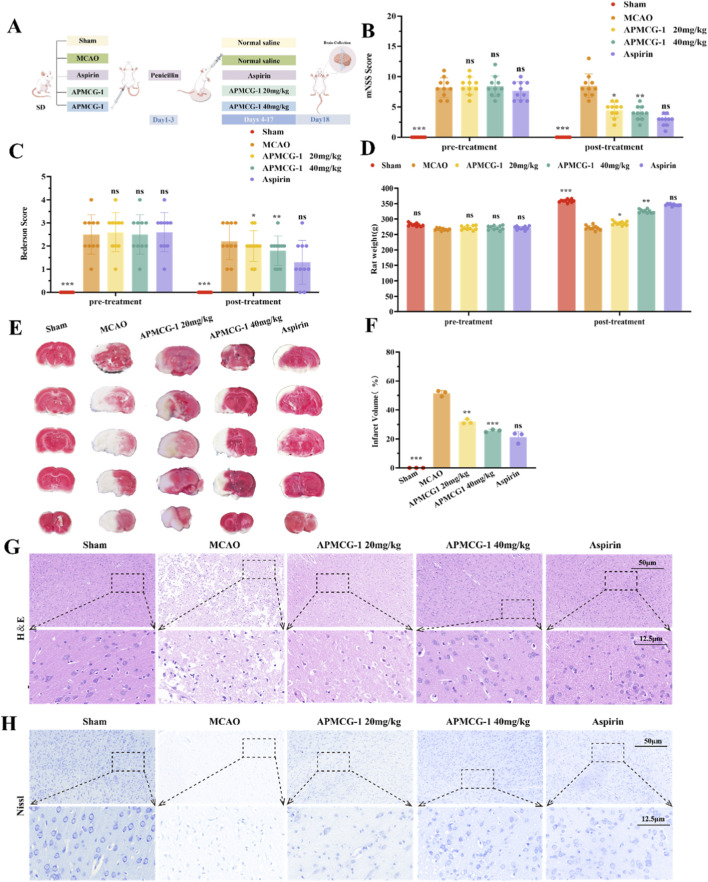
Pharmacological effects of APMCG-1 on MCAO Rats. **(A)** Flow chart of the experiment. **(B)** Neurological function scores of the mNSS in rats (n = 10). **(C)** Bederson neurological function scores in rats (n = 10). **(D)** Body weight of rats before and after treatment (n = 10). **(E, F)** TTC staining map and volume of cerebral infarction in rats. **(G)** H&E staining of the cerebral cortex of the rats, bar = 50 μm and 12.5 μm. **(H)** Nissl staining of the cerebral cortex of the rats, bar = 50 μm and 12.5 μm (Results other than neurological function scores are expressed as the means ± standard errors of three independent experiments, *P < 0.05, **P < 0.01, ***P < 0.001 compared with the model group. ^*P* < 0.05, ^^*P* < 0.01, ^^^*P* < 0.001 compared with the APMCG-1 40 mg/kg group. Statistical methods: one-way ANOVA followed by Tukey’s multiple comparisons test).

Rat Experiment Flowchart (Figure 1A). Comparison of neurological improvement between the treatment group and the model group before and after treatment. Before treatment, there was no significant difference in neurological dysfunction between the two groups. After treatment, APMCG-1 significantly improved neurological function compared to the MCAO group (Figures 1B,C). Rats in the MCAO group exhibited sustained weight loss, whereas APMCG-1 treatment promoted dose-dependent weight recovery. The 20 mg/kg group improved significantly, and the 40 mg/kg group showed comparable efficacy to aspirin, indicating equivalent health status restoration (Figure 1D). Through qualitative and quantitative analysis of TTC staining images, we observed more intuitively that both the high-dose APMCG-1 group (40 mg/kg) and the aspirin group demonstrated similar significant improvements in cerebral infarction volume compared to the model group (Figures 1E,F). Further H&E staining revealed marked nuclear shrinkage and deep staining in MCAO group neurons, accompanied by extensive vacuolated cytoplasm, widespread cellular disorganization and edema, and tangled neurofibrils (Figure 1G). Compared to the MCAO group, the APMCG-1 group showed marked improvement in cellular edema and vacuolation, with cell arrangement tending toward normalization and significant histopathological recovery. Nissl staining further revealed reduced Nissl bodies, neuronal shrinkage, central chromatin dissolution, and deeply stained, condensed nuclei in the MCAO group. However, in the APMCG-1-treated group and the aspirin group, the dissolution and disappearance of Nissl bodies were significantly improved after treatment, and neurons gradually regained their normal morphology (Figure 1H). These results indicate that APMCG-1 improves neurological function and protects against cortical tissue damage in MCAO rats. Its protective effects increase with concentration, with the optimal therapeutic effect observed at a dose of 40 mg/kg.

### 3.2 The link between stroke and oxidative stress, ferroptosis, and inflammation

**FIGURE 2:**
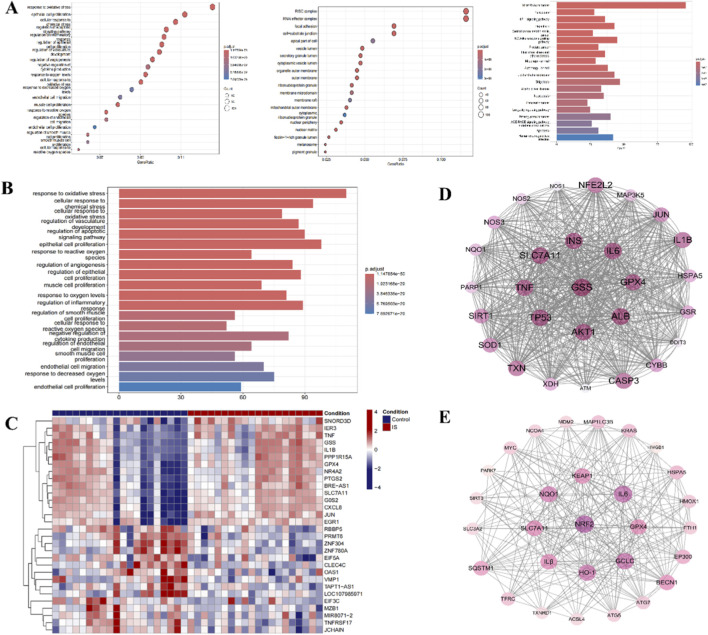
The link between stroke and oxidative stress, ferroptosis, and inflammation. **(A)** Bubble Plot of GO enrichment analysis: stroke-related oxidative stress, ferroptosis, and inflammation. **(B)** Bubble plot of GO enrichment of oxidative stress, inflammation, and ferroptosis. **(C)** Stroke-related gene heatmap. **(D)** Gene enrichment circle diagram of ferroptosis, oxidative stress, and inflammation. **(E)** Gene enrichment circle diagram for oxidative stress pathways.

Bioinformatic analysis of GEO datasets indicated that gene expression changes following stroke were enriched in pathways associated with oxidative stress, inflammation, and ferroptosis (Figures 2A–C). PPI network analysis suggested potential molecular crosstalk among these processes, with Nrf2 appearing as a highly connected node (Figures 2D,E). These *in silico* findings were interpreted as exploratory and hypothesis-generating, serving to inform the design of subsequent *in vitro* and *in vivo* experiments rather than to establish mechanistic conclusions. Experimental validation remains essential to confirm any causal relationships implied by the computational predictions.

### 3.3 APMCG-1 regulates oxidative stress, ferroptosis and inflammation levels *in vivo*

**FIGURE 3:**
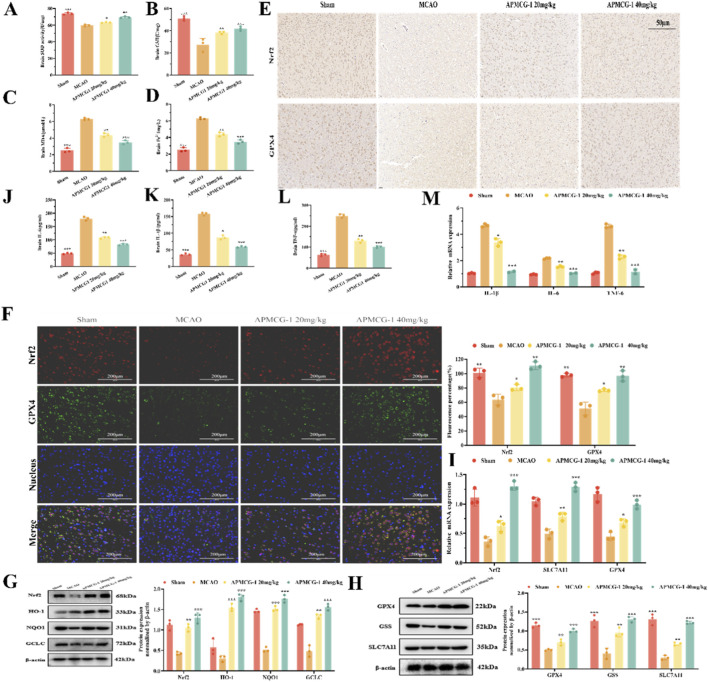
APMCG-1 regulates oxidative stress, ferroptosis and inflammation levels *in vivo*. **(A–D)** SOD, GSH, MDA and Fe^2+^ contents were assayed in the cortical tissue of the rat cortex. **(E)** Immunohistochemical examination of Nrf2 and GPX4 in rat cortical tissues. Scale bar = 50 μm. **(F)** Immunofluorescence staining of Nrf2 (red marker) and GPX4 (green marker) in the rat cortex by fluorescence microscopy and a fluorescence intensity histogram. Scale bar = 200 μm. **(G, H)** Rats cortices collected 14 days after APMCG-1 administration were examined for the expression of Nrf2, HO-1, NQO1, GCLC, GPX4, GSS, and SLC7A11 by Western blotting. **(I)** The mRNA levels of Nrf2, GPX4 and SLA7A11 in the cerebral cortex tissues of rats from different groups. **(J–L)** ELISA of the levels of the inflammatory factors IL-6, IL-1β and TNF-α in the rat cortex. **(M)** The mRNA levels of TNF-α, IL-6 and IL-1β in the cerebral cortex tissues of rats from different groups. The results are expressed as the means ± standard errors of three independent experiments; #*p* < 0.05, ##*p* < 0.01, and ###*p* < 0.001 compared with the control group. *p < 0.05, **p < 0.01, and ***p < 0.001 compared with the model group. Statistical methods: one-way ANOVA. Statistical methods: one-way ANOVA followed by Tukey’s multiple comparisons test).

Subsequently, we further tested the improvement in the cerebral cortex of MCAO rats following APMCG-1 treatment. SOD activity was higher in the APMCG-1-treated group than in the MCAO control group (Figure 3A), as well as GSH levels (Figure 3B). Simultaneously, compared with the MCAO group, APMCG-1 administration significantly reduced the accumulation of MDA and Fe^2+^ in brain tissue (Figures 3C,D). Therefore, we speculated that APMCG-1 ameliorated both oxidative stress and iron accumulation in the rat cortex following ischemia-reperfusion. Immunohistochemical results showed that Nrf2 and GPX4 positive staining gradually intensified with increasing drug concentrations (Figure 3E). Furthermore, through qualitative and quantitative analysis, immunofluorescent staining revealed that compared with the MCAO control group, the APMCG-1-treated group exhibited a dose-dependent increase in Nrf2 and GPX4 levels in the cerebral cortex (Figure 3F). Subsequent Western blot analysis of rat cortical tissue revealed that the four oxidative stress-related proteins Nrf2, HO-1, NQO1, and GCLC were significantly increased in a dose-dependent manner in APMCG-1-treated MCAO rats, while the three ferroptosis-related proteins GPX4, GSS (Glutathione Synthetase), and SLC7A11 were significantly higher than in the MCAO group, with the degree of increase being dose-dependent (Figures 3G,H). Furthermore, qPCR analysis revealed that the protein expression levels of Nrf2, GPX4, and SLC7A11 in rat cerebral cortex also showed a similar improvement trend with increasing APMCG-1 dosage (Figure 3I). Based on these findings, we could infer that APMCG-1 activated the Nrf2 pathway and its downstream proteins, as well as the expression of ferroptosis-related proteins, thereby alleviating oxidative stress and ferroptosis in the cortex of MCAO rats. Nrf2 pathway could also activate anti-inflammatory pathways, ELISA assay of inflammatory cytokines IL-6, IL-1β and TNF-α in rat cortical tissue showed significant reductions relative to the MCAO group with increasing APMCG-1 concentrations (Figures 3J–L), with qPCR yielding comparable results (Figure 3M). These findings indicate that cerebral ischemia-reperfusion induces oxidative stress, inflammation, and ferroptosis, suggesting their potential involvement in the pathological progression of ischemic brain injury. Importantly, APMCG-1 treatment effectively alleviated oxidative stress, inflammation, and ferroptosis in MCAO rats.

### 3.4 APMCG-1 regulates oxidative stress, ferroptosis, and inflammation levels *in vivo*, and inhibits therapeutic efficacy via the Nrf2 pathway

**FIGURE 4:**
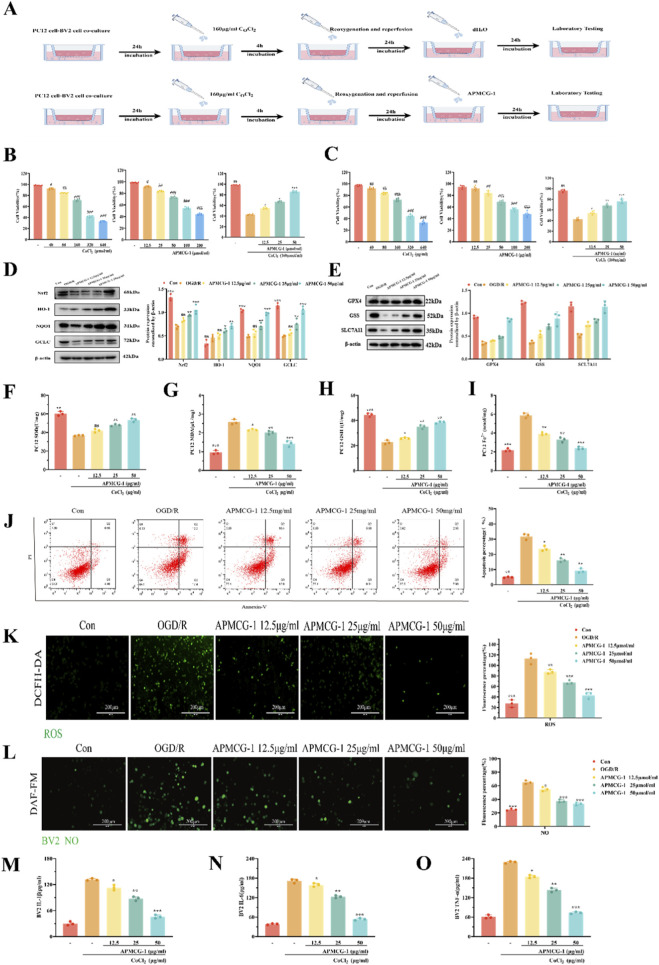
APMCG-1 regulates oxidative stress, ferroptosis, and inflammation levels *in vivo*. **(A)** Schematic diagram of the experimental procedure for the cell experiments. **(B)** Inducer concentration for PC12 cells, dose screening, and the effect of APMCG-1 on PC12 cell survival under glucose-deprived conditions. **(C)** Inducer concentration for BV2 cells, dose screening, and the effect of APMCG-1 on BV2 cell survival under glucose-deprived conditions. **(D, E)** The expression levels of Nrf2, HO-1, NQO1, GCLC, GPX4, GSS, and SLC7A11 in PC12 cells after APMCG-1 treatment were detected by Western blotting. **(F–I)** Detection of the MDA, SOD, GSH and Fe^2+^ contents in PC12 cells. **(J)** Flow scatter plots of PC12 cells used for the apoptotic assay. **(K)** Fluorescence microscopy of ROS in PC12 cells and the corresponding fluorescence intensity histogram. **(L)** NO staining of BV2 cells in the coculture system was observed under a fluorescence microscope, and fluorescence intensity analysis was performed. **(M–O)** ELISA analysis of the inflammatory factors IL-6, IL-1β and TNF-α in BV2 cells (The results are expressed as the means ± standard errors of three independent experiments; *p < 0.05, **p < 0.01 and ***p < 0.001 compared with the model group; and *p* < 0.05, andand *p* < 0.01 and andandand *p* < 0.001 compared with the APMCG-1 group. Statistical methods: one-way ANOVA followed by Tukey’s multiple comparisons test).

Cell Experiment Flowchart (Figure 4A). As shown in Figures 4B,C, we determined that the optimal CoCl_2_ concentration for PC12 and BV2 cells in glucose-free medium was 160 μg/mL when survival rates reached approximately 60% under inducer conditions. APMCG-1 > 50 μg/mL showed cytotoxicity. Thus, 12.5–50 μg/mL was selected as the effective non-toxic range. Compared with the OGD/R group, the cell survival rate in the 12.5 μg/mL APMCG-1 treatment group showed significant improvement (P < 0.05), and this survival rate progressively increased with higher APMCG-1 concentrations (P < 0.001 at 50 μg/mL). Therefore, we speculated that APMCG-1 exerts a therapeutic effect on cells following hypoxia and glucose deprivation. This range covers the estimated *in vivo* tissue exposure, ensuring translational relevance. We also quantitatively analyzed protein expression in PC12 cells across groups and found that the expression levels of oxidative stress and ferroptosis-related proteins (Nrf2, HO-1, NQO1, GCLC, GPX4, GSS, and SLC7A11) were significantly higher in the APMCG-1 group compared to the OGD/R group (Figures 4D,E). We similarly explored oxidative stress and ferroptosis markers in PC12 cells. SOD levels detected in the APMCG-1 group were higher than those in the OGD/R group (Figure 4F). Furthermore, MDA significantly accumulated in PC12 cells after oxidative stress (Figure 4G), while GSH levels decreased (Figure 4H). Compared with the OGD/R group, intracellular Fe^2+^ levels markedly decreased after APMCG-1 treatment (Figure 4I). As the APMCG-1 dose increased relative to the OGD/R group, the degree of apoptosis in PC12 cells also diminished, as demonstrated by flow cytometric analysis (see Figure 4J). ROS were detected in CoCl_2_-treated PC12 cells, but ROS fluorescence intensity significantly decreased with increasing APMCG-1 doses (Figure 4K). Damage to microglia triggers a series of inflammatory responses, thereby exacerbating neuroinflammation. Concurrently, NO staining revealed a decreasing trend in fluorescence intensity in BV2 cells with increasing APMCG-1 dose (Figure 4L). Furthermore, levels of inflammatory mediators IL-6, IL-1β, and TNF-α in BV2 cells were found to decrease in a dose-dependent manner with increasing APMCG-1 dose (Figures 4M–O). These results collectively indicate that APMCG-1 also alleviates oxidative stress and ferroptosis in OGD/R-induced PC12 cells. Furthermore, APMCG-1 mitigates inflammation in BV2 cells within the co-culture system.

**FIGURE 5:**
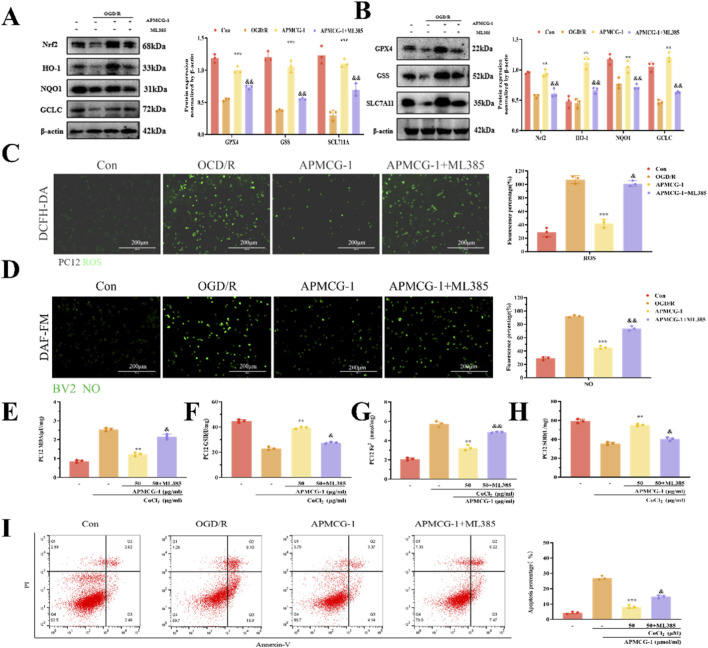
APMCG-1 protects neurons from hypoxia and glucose deprivation via the Nrf2 pathway. **(A, B)** WB detection of the effects of ML385 treatment on the expression levels of Nrf2, HO-1, NQO1, GCLC, SLC7A11, GPX4, and GSS proteins in PC12 cells. **(C)** ROS staining results of PC12 cells treated with ML385, along with their fluorescence intensity histograms. **(D)** NO staining results of BV2 cells treated with ML385, along with their fluorescence intensity histograms. **(E–H)** Detection of the MDA, SOD, GSH, and Fe^2+^ contents in the OGD/R-treated PC12 cell model following. **(I)** Flow cytometry results of PC12 cells treated with ML385, along with their apoptosis proportion histograms (The results are expressed as the means ± standard errors of three independent experiments; *p < 0.05, **p < 0.01 and ***p < 0.001 compared with the model group; and *p* < 0.05, andand *p* < 0.01 and andandand *p* < 0.001 compared with the APMCG-1 group. Statistical methods: one-way ANOVA followed by Tukey’s multiple comparisons test).

To further demonstrate that APMCG-1 exerts neuroprotective effects via the Nrf2 pathway, we added ML385 for specific direct inhibition of Nrf2 transcriptional activity and conducted further discussion and analysis of the relevant factors. When ML385 was added to the APMCG-1 group, limiting the expression of HO-1, NQO1, GCLC, GPX4, GSS, and SLC7A11 (Figures 5A,B). Additionally, ROS staining analysis revealed that PC12 cells produced higher levels of NO in the presence of the inhibitor (Figure 5C). Moreover, NO staining analysis revealed that BV2 cells produced higher levels of nitric oxide in the presence of the inhibitor (Figure 5D). The ability of APMCG-1 to improve BV2 cell inflammation was inhibited. Concurrently, compared to the APMCG-1 group, PC12 cells exhibited reduced total antioxidant capacity and levels of Fe^2+^ in cells, decreased SOD and GSH activity, and increased MDA and ferrous ion levels (Figures 5E–H). Moreover, the degree of apoptosis in PC12 cells was also reduced. Flow cytometry analysis revealed that the level of apoptosis in PC12 cells from the APMCG-1 group was significantly lower than that in the OGD/R group, whereas the level of apoptosis in PC12 cells from the inhibitor group was markedly higher than that in the APMCG-1 group (Figure 5I). We therefore infer that under the inhibitory effect of ML385, the expression of Nrf2 pathway-related proteins is suppressed, thereby inhibiting the therapeutic effect of APMCG-1 on damaged neurons. In summary, these results indicate that APMCG-1 alleviates oxidative stress, ferroptosis, and inflammatory responses in PC12 and BV2 cells following OGD/R via the Nrf2 pathway.

### 3.5 APMCG-1 could also inhibit therapeutic efficacy *in vivo* via the Nrf2 pathway

**FIGURE 6:**
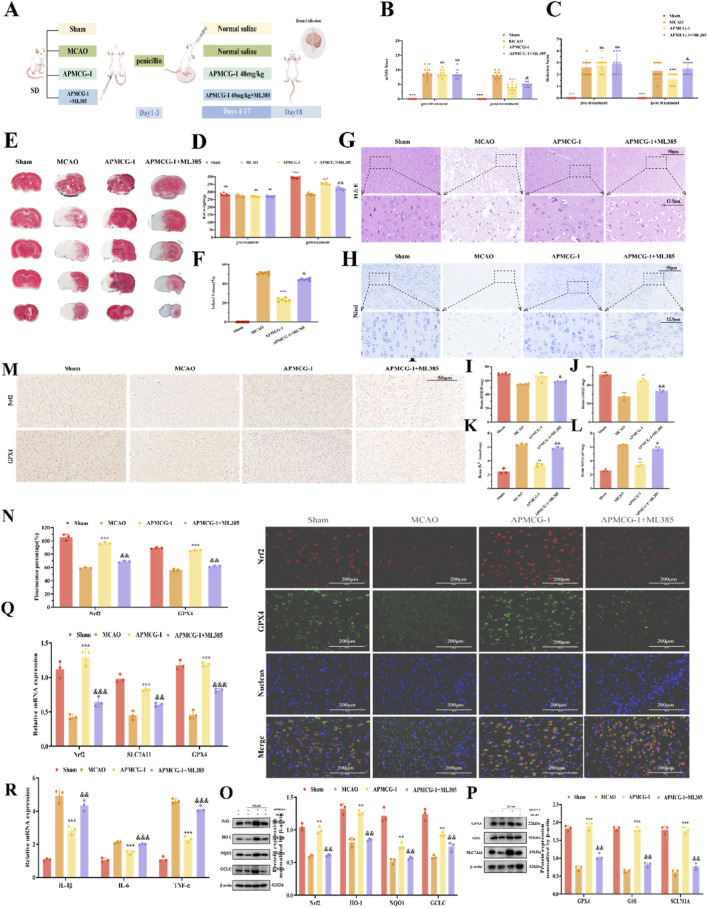
APMCG-1 can also inhibit therapeutic efficacy *in vivo* via the Nrf2 pathway. **(A)** Schematic diagram of the experimental procedure for MCAO rat experiments involving ML385 treatment. **(B)** mNSS for evaluating neurological function in the rats (n = 10). **(C)** Bederson neurological function scores of the rats (n = 10). **(D)** Body weight of rats before and after treatment (n = 10). **(E, F)** TTC staining map and volume of cerebral infarction in rats. **(G, H)** HE staining and Nissl staining of the rat cerebral cortex. Scale bar = 50 μm and 12.5 μm. **(I–L)** SOD, GSH, MDA and Fe^2+^ contents were assayed in the cortical tissue of the rat cortex under the influence of ML385. **(M)** Immunohistochemical examination of Nrf2 and GPX4 in rat cortical tissues after the addition of ML385 and its fluorescence intensity histogram. Scale bar = 50 μm. **(N)** The expression of Nrf2 and GPX4in the cortices of the rats treated with ML385 was observed by fluorescence microscopy, and fluorescence intensity analysis was performed. Scale bar = 200 μm. **(O, P)** Representative WB bands and quantitative analysis of the effects of ML385 treatment on the expression of Nrf2, HO-1 NQO1, GCLC, SLC7A11, GSS and GPX4 in rat cortices. **(Q, R)** The mRNA levels of Nrf2, SLC7A11, GPX4, IL-6, IL-1β, and TNF-α in the brain tissues of rat cortices from different groups (The results are expressed as the means ± standard errors of three independent experiments; *p < 0.05, **p < 0.01 and ***p < 0.001 compared with the model group; and *p* < 0.05, andand *p* < 0.01 and andandand *p* < 0.001 compared with the APMCG-1 group. Statistical methods: one-way ANOVA followed by Tukey’s multiple comparisons test).

Rat Experiment Flowchart (Figure 6A). Neurological function test results and body weight changes indicate that the ML385 inhibitor group showed less improvement in neurological function and body weight compared to the APMCG-1 group (Figures 6B–D). In the ML385 inhibitor group, TTC staining, HE staining, and Nissl staining results indicated that the inhibitor group demonstrated less improvement in cortical lesions compared to the APMCG-1 group (Figures 6E–H). Compared with the APMCG-1 group, the inhibitor group exhibited reduced levels of SOD and GSH in rats (Figures 6I,J), while MDA and Fe^2+^ levels also showed significant differences (Figures 6K,L). In immunohistochemistry, Nrf2 and GPX4 showed faint positive staining in the inhibitor group (Figure 6M). Meanwhile, immunofluorescence analyses revealed that Nrf2 and GPX4 expression levels in the cerebral cortex of rats in the inhibitor group were suppressed, showing significant differences compared to the APMCG-1 group (Figure 6N). We further investigated the expression of Nrf2 pathway and ferroptosis-related proteins in the rat cortex following ML385 treatment. We found that the expression levels of Nrf2, HO-1, NQO1, GCLC, GPX4, GSS, and SLC7A11 were all suppressed in the inhibitor group (Figures 6O,P). Concurrently, mRNA expression of Nrf2, GPX4, SLC7A11, IL-6, IL-1β, and TNF-α was also inhibited (Figures 6Q,R). The addition of the ML385 inhibitor directly suppressed the expression of Nrf2 and its pathway-associated proteins, thereby inhibiting the amelioration of oxidative stress, ferroptosis, and inflammation in the cerebral cortex of MCAO rats by APMCG-1. Therefore, based on the above experimental results, we speculate that APMCG-1 might alleviate oxidative stress, ferroptosis, and inflammatory responses through the Nrf2 pathway, thereby improving cortical damage in rats following ischemia-reperfusion injury.

## 4 Discussion

Ginseng has historically played a role in the therapeutic management of IS. The efficacy with which it promotes blood circulation as well as qi has been thoroughly documented. Ginseng is a botanical material. It contains three active ingredients: ginsenosides, gintonin, and polysaccharides (Zhao et al., 2022). Currently, isolation procedures typically target only one of these three components. The remaining components are then disposed of as waste. These three main components have been extensively utilized in the therapy of numerous diseases (Ling et al., 2024; Zhou et al., 2024; Wang et al., 2021). However, the therapeutic potential of non-saponin components, particularly glycopeptides from processing residue, remains underexplored.

A novel glycopeptide from mountain-cultivated ginseng residue known as APMCG-1 has been shown to exert protective effects on IS. For example, our previous study proposed that APMCG-1 exhibited antioxidant and antiapoptotic properties in IS rat and zebrafish models (He et al., 2024). The present study builds upon this foundation by further revealing Nrf2-dependent ferroptosis inhibition as a novel protective mechanism, expanding the multi-target action mode of APMCG-1.

IS represents the second most prevalent cause of mortality and disability on a global scale. This condition frequently results in motor and cognitive impairments (Zhang et al., 2025). Damage to the cerebral cortex after IS results in impaired mobility and severe disability. As such, it is considered one of the world’s most challenging diseases. IS is characterized by cerebral blood flow interruption followed by therapeutic or spontaneous reperfusion, which triggers ischemia-reperfusion injury. This process involves initial ischemic damage and subsequent reperfusion-mediated secondary injury driven by oxidative stress, ferroptosis, and neuroinflammation (Zhang et al., 2024).

Brain tissue is rich in phospholipids, and lipid peroxidation is a prominent feature of brain injury. Studies indicate iron ion accumulation and iron-dependent lipid peroxidation occur during the pathological process of ischemic brain injury (Li X. et al., 2025). Extensive studies indicate that ferroptosis might contribute to worsened neurological outcomes following ischemic brain injury (Zhu et al., 2024; Tang and Kroemer, 2020; Song et al., 2025). In our study, we investigated the neuroprotective effects of APMCG-1 in a stroke model and its potential mechanisms. Data demonstrated that APMCG-1 significantly reduced neurological scores, indicating its efficacy in alleviating post-stroke neurological dysfunction. TTC results revealed that APMCG-1 significantly reduced the size of the cerebral infarction area, indicating that APMCG-1 intervention helped mitigate brain tissue damage. Furthermore, H&E and Nissl staining showed that APMCG-1 significantly restored neuronal morphology and increased the number of neurons in the treatment group. The highest efficacy was observed at the 40 mg/kg dose, comparable to the clinical anti-inflammatory drug aspirin (Wu et al., 2019). These findings suggests that APMCG-1 could effectively alleviate neuronal damage.

The interaction between inflammation and oxidative stress forms a vicious cycle that continuously exacerbates the severity of neuropathological damage (Sun et al., 2023). Concurrently, research indicates that oxidative stress and inflammatory processes are closely associated with ferroptosis (Astudillo et al., 2023), a fact that further intensifies this pathological state. Therefore, effective treatment of ischemia-reperfusion injury hinges on simultaneously inhibiting ferroptosis, alleviating oxidative stress, and suppressing pro-inflammatory factor release. APMCG-1 significantly suppressed levels of inflammatory cytokines (IL-6, IL-1β, and TNF-α) and the oxidative stress marker MDA, while markedly increasing levels of the antioxidant stress markers GSH and SOD. This indicates that APMCG-1 effectively alleviates oxidative stress and neuroinflammation induced by MCAO. Similarly, APMCG-1 significantly reduced ferrous iron in the cortex or cells accumulation, attenuated lipid peroxidation MDA, increased GPX4 protein expression, restored GSH, and upregulated cystine/glutamate antiporter SLC7A11 and GSS. These coordinated changes indicated that ferroptosis was suppressed. Furthermore, the effects of APMCG-1 were blocked by ML385, suggesting that APMCG-1 might suppress oxidative stress, ferroptosis and neuroinflammation by activating Nrf2.

Extremely severe neuroinflammation has been documented as a subsequent occurrence of cerebrovascular accidents (Yang et al., 2019). Microglia, the primary immune cells within the central nervous system (CNS), play critical roles in the progression of stroke (Liang et al., 2023). The activation of microglia instigates an inflammatory response, which can lead to excessive neuroinflammation (Kwon, 2022). This, in turn, has the potential to exacerbate neuronal and glial cell damage, impede brain repair, and hinder functional recovery (Tang et al., 2021). The present study demonstrated that APMCG-1 not only protected neurons but also suppressed microglial activation, reducing IL-1β, TNF-α, and IL-6 via the Nrf2/HO-1 pathway inflammation, achieving neuro-immune dual protection. Using a PC12 and BV2 cell coculture system, APMCG-1 alleviated oxidative stress and inhibited ferroptosis in PC12 cells following hypoxia-glucose deprivation, while simultaneously mitigating inflammation in BV2 cells. This dual-cell-type protection highlighted the therapeutic advantage of targeting Nrf2 over single-pathway interventions.

**FIGURE 7:**
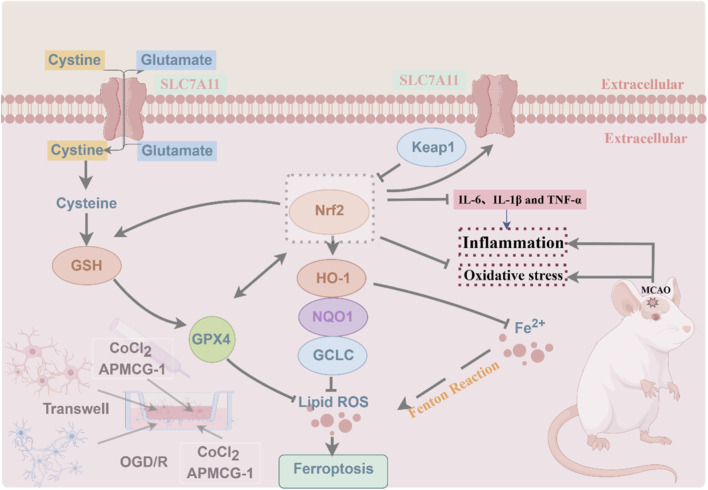
Schematic diagram of the Nrf2 pathway mechanism.

Extensive research indicates that the Nrf2 signaling pathway plays a crucial regulatory role in modulating lipid peroxidation and alleviating inflammation (Wang et al., 2022). Within the paradigm of antioxidant stress, Nrf2 functions as a central regulator of the antioxidant response, exerting its anti-ferroptotic effects by modulating target gene expression (Jiang et al., 2023). Upon entering the nucleus, Nrf2 binds to antioxidant response elements to activate the expression of antioxidant defense-related genes (including HO-1, GCLC, and NQO1) (Jiang et al., 2020). SLC7A11 is essential for GSH synthesis. Inhibition of SLC7A11 disrupts cellular redox homeostasis, leading to ferroptosis. GPX4 degrades small-molecule peroxides and lipid peroxides while inhibiting lipid peroxidation. GSH serves as the primary substrate for GPX4; reduced GPX4 activity due to GSH depletion fails to eliminate harmful lipid peroxides (Li et al., 2021; Yang et al., 2024; Cui et al., 2022). Therefore, GPX4, SLC7A11, and GSS constitute key molecular components of the Nrf2-mediated ferroptosis defense network. To clarify the mechanism by which APMCG-1 inhibits ferroptosis, we analyzed the expression of Nrf2 and downstream ferroptosis-related proteins. The data revealed that APMCG-1 significantly upregulated the expression of Nrf2, HO-1, NQO1, GCLC, SLC7A11, and GSS, whereas ML385 inhibited the influence of APMCG-1 on these proteins, suggesting that APMCG-1 might inhibits ferroptosis in stroke by targeting the Nrf2 pathway. Moreover, in both *in vitro* and *in vivo* ischemia-reperfusion models, the neuroprotective effect of APMCG-1 became more pronounced with increasing concentrations of the drug. In conclusion, this study suggests that APMCG-1 plays a role in protecting neurological function in stroke and that the Nrf2 pathway is a potential target of APMCG-1. This protective effect might be related to the inhibition of oxidative stress, neuroinflammation and ferroptosis (Figure 7).

This study has several limitations. First, only male rats were used to avoid potential interference from estrogen and progesterone fluctuations associated with the female estrous cycle on iron metabolism homeostasis, antioxidant defense, and inflammatory responses; therefore, extrapolation of these findings to female populations should be interpreted with caution. Future studies should include female animals or systematically compare sex differences in the neuroprotective effects of APMCG-1 under conditions of controlled estrous cycles. Secondly, we lack experimental methods to detect mitochondrial morphological changes, preventing us from providing more direct evidence for the occurrence of ferroptosis. Future studies should incorporate transmission electron microscopy to observe mitochondrial morphological alterations. Thirdly, we acknowledge that proximal activation events—including Nrf2-Keap1 dissociation, nuclear translocation, and direct ARE binding—were not assessed. Future studies employing Keap1 knockout, Nrf2 luciferase reporter assays, or proximity ligation assays for Nrf2-Keap1 interaction are required to elucidate the proximal mechanism.

## 5 Conclusion

In summary, this study demonstrates that APMCG-1 significantly attenuates cerebral ischemia-induced injury *in vivo* and *in vitro* through Nrf2-dependent suppression of ferroptosis, alleviation of oxidative stress, and mitigation of neuroinflammation. These data offer novel insights into the mechanisms of ferroptosis and the Nrf2 pathway in IS, expanding the pharmacological role of ginseng beyond traditional saponin components.

## Data Availability

The original contributions presented in the study are included in the article/supplementary material, further inquiries can be directed to the corresponding author.
